# Transforming the field: the role of academic health centers in promoting and sustaining equity based community engaged research

**DOI:** 10.3389/fpubh.2023.1111779

**Published:** 2023-06-22

**Authors:** Shannon Sanchez-Youngman, Prajakta Adsul, Amber Gonzales, Elizabeth Dickson, Katie Myers, Christina Alaniz, Nina Wallerstein

**Affiliations:** ^1^College of Population Health, University of New Mexico, Albuquerque, NM, United States; ^2^Department of Internal Medicine, University of New Mexico, Albuquerque, NM, United States; ^3^College of Nursing, University of New Mexico, Albuquerque, NM, United States; ^4^School of Medicine, University of New Mexico, Albuquerque, NM, United States

**Keywords:** institutional trustworthiness, community-engaged institutions, facilitative leadership, power sharing, empowerment

## Abstract

Community-based participatory research (CBPR) and community engaged research (CEnR) are key to promoting community and patient engagement in actionable evidence-based strategies to improve research for health equity. Rapid growth of CBPR/CEnR research projects have led to the broad adoption of partnering principles in community-academic partnerships and among some health and academic organizations. Yet, transformation of principles into best practices that foster trust, shared power, and equity outcomes still remain fragmented, are dependent on individuals with long term projects, or are non-existent. This paper describes how we designed our Engage for Equity PLUS intervention that leverages the leadership and membership of champion teams (including community-engaged faculty, community partners and patient advocates) to improve organizational policies and practices to support equity based CBPR/CEnR. This article describes the feasibility and preliminary findings from engaging champion teams from three very different academic health centers. We reflect on the learnings from Engage for Equity PLUS; the adaptation of the intervention design and implementation, including the development of a new institutional assessment using mixed research methods; and our organizational theory of change. In summary, our design and preliminary data from the three academic health centers provide support for new attention to the role of institutional practices and processes needed to sustain equity-based patient and community-engaged research and CBPR and transform the field.

## 1. Introduction

With the dual pandemics of COVID and structural racism, which have devastated communities of color and other vulnerable communities, community based participatory research (CBPR) and community engaged research (CEnR) have never been more important for health equity goals (1, 2). These two terms have signified a range of strategies for community stakeholders engaging as partners in different stages of the research. CEnR consists of a continuum of minimum engagement through outreach, to greater shared leadership (3); with CBPR focusing on transforming power imbalances to elevate community priorities and community-driven research leadership (4). The Patient Centered Outcomes Research Institute (PCORI), as a federal funding agency launched in 2010, has added engagement of patients and patient advocates as key community partners. These efforts have led to a broad adoption of principles of engagement, as a motivating force behind grassroots community health interventions to more traditional clinical medicine interventions led by academic physicians. The clinical and translational science awards (CTSAs) since 2006 have reinforced community engagement in more than 60 academic health centers, adding to other NIH-funded translational equity centers, CDC-funded prevention research centers, and newer funding for comprehensive cancer centers, all of which have increasingly required community engagement cores (CECs). The field itself has grown beyond an emphasis on principles and practices, towards a focus on health and health equity outcomes that are promoted by community participation in all stages of research among other “best” practices (5–7). A new National Academy of Medicine engagement model has reinforced the importance of outcomes, i.e., strengthened partnerships, expanded knowledge, improved health and health care programs and policies, and thriving communities (7).

Despite this maturation of the field, significant gaps remain in the uptake and diffusion of a PCORI/CEnR/CBPR framework across institutional settings and federally-and foundation-funded grant initiatives. Even more importantly, practitioners and long-term leaders in the field increasingly recognize that research partnerships cannot singlehandedly drive health equity outcomes, nor can they support sustainable long term, health equity efforts without more cohesive and structured institutional support. A key learning in national dialogues is that partnered health mobilization efforts should exist beyond grant-funded cycles to reach successes in health equity outcomes (8).

Thus, examining the role of institutional Academic Health Center (AHC) contexts of research and research support becomes essential, including how they interact with communities and sustain (or not) efforts to ameliorate health disparities. Barriers to working with communities noted in the literature have included ongoing distrust by community members of AHCs, with demands for greater “trustworthiness” of these institutions, including the need to pair engagement strategies with anti-racism diversity, equity and inclusion efforts (9–11). Recent uncovering of realities of fiscal and administrative contextual barriers within AHCs showcase how they have not been responsive to the needs of community organizations, patient advocacy groups, tribes, and other partners (11, 12).

This paper describes the rationale for the need for institutional changes in research contexts at the institutional level; and presents our intervention, called Engage for Equity PLUS, aimed at transforming institutional policies, processes and norms. We present our logic model, theory of change, design and strategies; and offer cross-institutional preliminary results that highlight the potential for institutions to become more community-responsive and trustworthy enough to make a difference in health equity over the long-term.

With PCORI engagement funding, the University of New Mexico’s Center for Participatory Research (UNM-CPR) has been implementing “Engage for Equity (E2) PLUS” with Morehouse School of Medicine, Stanford School of Medicine and Cancer Institute, and Fred Hutchinson/University of Washington Cancer Consortium since 2021. Engage for Equity PLUS emerged as a scaled-up strategy for academic health centers after 17 years of NIH funding of “Engage for Equity” (E2), by UNM-CPR with national partners, to identify engagement best practices at the project level associated with health and health equity outcomes.

Engage for Equity had earlier produced a CBPR conceptual model, with four domains (of contexts, partnering processes, intervention and research design actions, and intermediate and long-term outcomes) (13); tested and validated measures of practices and outcomes within each domain with more than 400 diverse federally-funded community-academic research partnerships (14, 15); identified and tested the E2 intervention of workshops and use of collective reflection tools to strengthen partnerships (16); and modeled pathways of how engagement practices contribute to outcomes (7) such as trust and other relationship strategies (17); and co-governance structures (18).

While producing outcomes at the project level, the Engage for Equity team realized they needed to implement the intervention at the institutional level as the next step for Academic Health Centers to become more effective at promoting and sustaining cross-sector collaborations between universities and community stakeholders. Using a mixed methods engagement approach, Engage for Equity PLUS study had three primary aims:

1. To assess institutional contextual factors (i.e., capacity, structures, process, and commitment to equity-based engagement) in three distinct Academic Health Centers to promote and sustain patient and community engaged research
2. To test the feasibility of the E2 PLUS intervention, applying E2 workshops and collective reflection tools, with a new added component of institutional champion teams as facilitative leaders to advocate for changes in academic health centers; and
3. To develop a mutual learning community of practice among champion teams from the three institutions.

The context of institutional barriers to community engaged research is described below, followed by a full description of the E2 PLUS intervention.

## 2. Barriers to support, increase and sustain CEnR research

Over the last several years, scholarship has revealed multiple challenges and barriers for academic health centers (AHCs) to more systematically support, increase, and sustain CEnR research. Three key challenges have emerged: the ongoing and rising public distrust of academic institutions (9–11); the reality of institutional policies, practices and norms that favor AHCs’ interests in garnering funding to support internal research infrastructures rather than sharing power with community (19); and the increasing need to develop and test multi-level interventions to transform these power imbalances (20, 21).

Challenge 1: CBPR research practices may have increased trust at the partnership level, but community stakeholders continue to report ongoing distrust and lack of institutional trustworthiness in academic health centers. Despite the growth of community members engaged as research co-designers, implementers, and in project advisory committees or CTSA community boards, trust still remains a core issue with the need to articulate what trust means at the institutional level, beyond participating in research trials (22). There is ample evidence that community members participating in AHC efforts continue to demonstrate concerns of being undervalued, lacking perceived power, receiving inadequate resources, and being relegated to advisory committee roles, without decision-making authority. Studies have shown, for example, that only 10% of CTSA institutions invite community members to participate in core areas of research (23); that community members identify cultural disconnects between AHCs and community, such as lack of success metrics other than academic publications and lack of funding for community partners (10); and that contextual barriers make it difficult for community members to participate in research, including undocumented legal status, homelessness, or having little political power in their lives (24).

Institutional trustworthiness is also being regarded as the most commonly cited reason for lack of participation of minorities in research trials (25). The COVID-19 pandemic has exacerbated institutional distrust, with communities of color suffering higher mortality rates, which tragically has reproduced traumas from previous histories with research and medical institutions (26). Academic health centers continue to be charged with having hierarchies of structural racism, systems of inequitable care or inequitable distribution of resources (2). Our E2 PLUS intervention explicitly tackles these trends by incorporating strategies to improve the trustworthiness and accountability of AHCs to the communities they seek to engage in research and health equity efforts.

Challenge 2: institutional and structural forms of power limit the ability of CEnR researchers and community partners to execute transformational research. Grant funded and institutionally supported CBPR initiatives have made incremental strides in moving from purely investigator-controlled initiatives toward promoting research practices that foster collaboration with communities in project-level research. Much of this work has focused on fostering research partnerships that promote cooperative relationships (27), shared governance (18, 28), increased community capacity (21, 29), cultural revitalization (30, 31), inclusion and belonging, and community resilience (32–34). At the project level, these partnerships have led to capacity outcomes such as increases in community involvement in all phases of health research, shared power, synergistic partnerships between researchers and community stakeholders, towards longer term health and health equity goals (5).

While helpful, this inward focus has promoted a degree of instrumentalism in the field with much scholarship focusing on the ‘ingredients’ needed to achieve more effective engagement within discrete research partnerships funded to impact categorical health outcomes (21, 29). While important, this approach underplays how contextual challenges impact the transparency, commitment, accountability, and efficiency of multiple stakeholders to advance strategies that achieve health equity transformation (35–37). Many of these challenges can be linked to power asymmetries that are manifested in multiple ways in PCOR/CEnR research and practice (21, 36, 38–41). Using a limiting power framework, Popay (21) and her colleagues have described multiple forms of power that inhibit community empowerment as a route to greater health equity.

Key among them is institutional power, which is exercised through organizational rules, procedures, and norms. Within academic health center bureaucracies, institutional power imbalances are often manifested in draconian management expectations that require community stakeholders to interface with fragmented and impenetrable fiscal, research, and contracting systems. For example, Carter-Edwards and her colleagues (2021) have documented multiple procedural and policy barriers inhibiting effective collaboration within CTSAs. They find that both principal investigators and community stakeholders lack familiarity with unclear fiscal and grant administration processes, community partners are burdened by challenges in navigating institutional fiscal management processes that remain un-adapted to meet the needs of community organizations, and there is a dearth of organizational practices that lead to appropriate management of budgets and timely compensation of community partners (12). Our E2 intervention starts from a deep dive into these barriers and contexts that need to be unmasked as a first step towards institutional transformation.

Challenge 3: academic health centers struggle to collaborate with community stakeholders in their long-term mobilization and organizing efforts to advance health equity through sustained multilevel interventions, policy advocacy, and transformational changes outside of academic settings. Advancing health equity has proven to be a complex and long-term endeavor that requires collaboration and power-sharing between policy makers, researchers, public and private organizations, policy makers, elected officials, administrators, place-based constituencies, patient advocates, and identity-based communities. Multi-sector partnerships striving to improve health equity do not start and end with grant funded research. They require ongoing collaborations at multiple levels to deepen and sustain innovative solutions that address the social determinants of health and structural racism. For example, patient movements and coalitions have demanded that academic health centers participate in their longer-term efforts to address health care systems issues related to chronic disease, cancer survivorship, and preventative screening. CBPR has been cited as a key strategy to promote longer-term health equity because it embraces empowering research processes that have been shown to contribute to the capabilities of patients and communities to exercise control over decisions and actions that influence their lives and health (20).

In addition, while funders acknowledge structural determinants and health care systems barriers, the requirement that health interventions demonstrate effectiveness in changing patient and individual level health outcomes has produced interventions that tend to privilege clinical and community interventions that aim to increase positive psycho-social outcomes *within* disadvantaged communities and patient populations. Common examples include intervention efforts that promote community resilience, healthy behaviors, and cultural recognition (21, 30). This individual focus on placed-based groups often neglects the social and political determinants of health, such as legacies of racism and settler colonialism, leaving systems of power and privilege intact (21).

Consequently, these approaches have succeeded in supporting marginalized communities to *adapt* to conditions of structural racism, disinvestment, and structural violence without fundamentally changing them (42–44). In addition to funding constraints, CEnR initiatives continue to struggle with transforming power into concrete practices (45). Returning to Wallerstein’s (46) argument that power must be dissected to achieve collective empowerment, we argue that we must re-direct our attention towards multi-level interventions that guide us in “how to analyze and understand changing configurations of power” (39) in order to achieve longer term health equity outcomes. In short, CEnR initiatives must deconstruct how power operates in multiples contexts in order to identify viable solutions for change.

## 3. Methods and E2 PLUS process design

E2 PLUS aims to address these three barriers by expanding the evidence-based E2 intervention to test the feasibility of institutional engagement strategies designed to produce cultural shifts and structural changes with three participating academic health centers. Each site differed in its level of equity-based PCOR/CEnR based on their history and research priorities. Morehouse School of Medicine is a HBCU born from a need to fight racial health disparities in Atlanta, GA with a long history of collaborating with community leaders and local community based organizations to address health disparities. Fred Hutchinson/University of Washington Cancer Consortium is a designated comprehensive cancer center with a new consortium made up of three institutions (University of Washington, Fred Hutchinson Cancer Research Center, and Seattle Children’s Hospital) seeking to bring together their individual histories of community engaged cancer research. Stanford is a highly prestigious private institution, which has centered its health research efforts on innovations in basic and clinical science.

**Figure 1:**
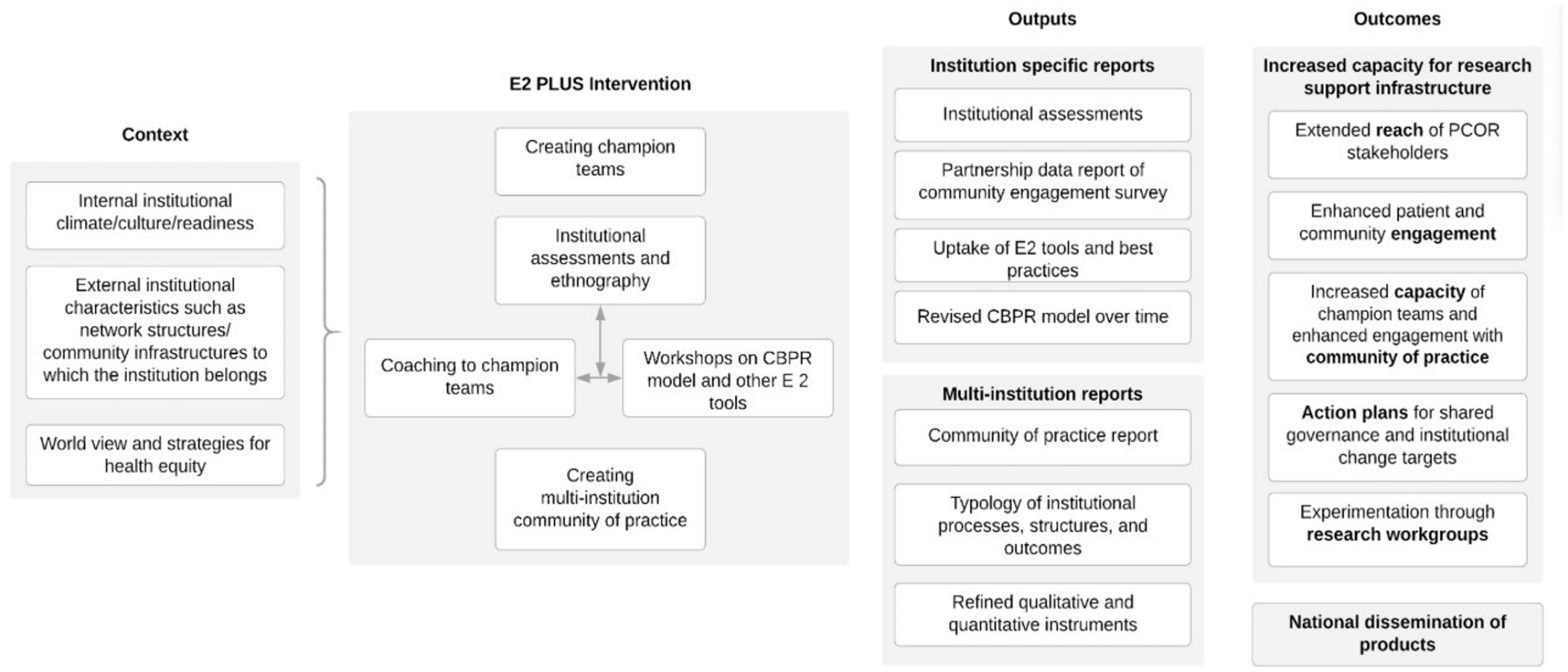
Engage for equity logic model.

Building from theories of institutional change, collaborative governance, and models of organizational engagement (36, 47) E2 PLUS as an institutional intervention, added to its workshops and collective reflection tools, a new component, the role of champion teams as facilitative leaders to advocate for reshaping institutional research infrastructures towards equity-centered PCOR and CEnR. Figure 1 describes the overall logic of the intervention including the primary outcomes, with E2 PLUS strategies described below.

Strategy 1: establish champion teams and provide coaching for them to implement facilitative leadership practices. In the first year of the project, the research team collaborated with project leads to establish champion teams consisting of 6–12 members including academic health center leaders, faculty, community partners, and patient advocates invested in PCOR/CEnR at their institution. Champion teams met monthly for coaching with the UNM-CPR team, using Zoom for the online intervention in the first intensive year of workshops and data collection; and have met less often in year two, depending on the chosen strategies. UNM coaching included training in use of the Engage for Equity tools through workshops, providing qualitative and quantitative data of institutional barriers and facilitators, and supporting the development of action strategies and working groups to advocate for specific changes. Our coaching intervention has been designed to support champion teams to identify advantage points for institutional change and to engage in rapid-cycle testing of actions as they develop into facilitative leaders. Building from our previous work, we have used an iterative reflection approach with the teams to strategize, prioritize, and plan next steps.

Strategy 2: provide workshops with E2 tools. In the first year of the project, the UNM team conducted two virtual workshops for 25–35 stakeholders that included champion team members, community engagement staff and leaders, researchers, patients/patient advocates, community advocates and leaders. Workshops offered interactive learning activities based on previously validated E2 tools that guided participants through collective reflection and strategic planning for institutional change. The first workshop started with the E2 Tool, the *Institutional River of Life,* which engaged stakeholders to collaboratively construct their engagement history, or a visual metaphor of shared historical and community experiences, grounding participants in their own contexts (48). This was followed by the E2 Tool, *Visioning with the CBPR Model*, for stakeholders to develop their first collective strategic action plan, using the CBPR model to envision desired outcomes, needed additional partners, and actions to reach outcomes. In the second workshop, a synthesis of qualitative and quantitative data was presented followed by dialogue (in breakout rooms) to re-Vision with the CBPR Model their action plans and working groups for changes in institutional processes and policies. Building from our previous workshop interventions with partnered projects, the E2 tools remain grounded in the Freirean praxis of iterative cycles of collective reflection and action and create momentum to push for change at the institutional level (49).

Strategy 3: collect and use institutional data for advocacy. After the first workshop, the research team collected quantitative and qualitative data from leader interviews and patient/community member focus groups to assess the extent to which institutional policies, procedures, and norms support PCOR/CEnR research and stakeholder engagement at the institutional level. The UNM team regularly collected, cleaned, organized, and shared data with each of the three academic health centers describing institutional facilitators and barriers from the perspective of different stakeholders. Recommendations from qualitative data, together with institutional survey baseline survey results, were presented to the wider group at the second workshop to solidify working groups for collectively leveraging actions for change over the second year of the intervention. These institutional assessments were designed to reveal differing stakeholder perspectives and tensions identified in the data to promote critical reflection on next steps, with recommendations from community members/patient advocates given high priority.

Strategy 4: support community and patient advocate power. Throughout the intervention, community/patient advocate partners were given opportunities to exercise their power, through participating on champion teams, through prioritizing their perspectives from the patient/community focus group, and from separate meetings in workshop breakout rooms to provide a safe place to interact and document their recommendations.

Strategy 5: co-create a community of practice with the three participating institutions. In the first year, we invited the academic and community/patient co-leads from each champion team to join quarterly, multi-site, Zoom calls to share their interests and concerns in strengthening research support for patient and community engagement. These quarterly meetings were designed to create a community of practice (50, 51) to establish norms of sharing across institutions their actions, goals, and desired outcomes. As part of the community of practice, teams shared their organizational Rivers of Life and CBPR Model Visioning as visualizations of their process; and have had the opportunity through a panel, at the Action Research Network of the Americas conference after the first year in June 2022, to share their data from the institutional assessments which provided a collective understanding of their institutional barriers to strengthening their engagement support infrastructures, as well as their shared and unique assets and strategies for change.

### 3.1. Theory of change

**Figure 2:**
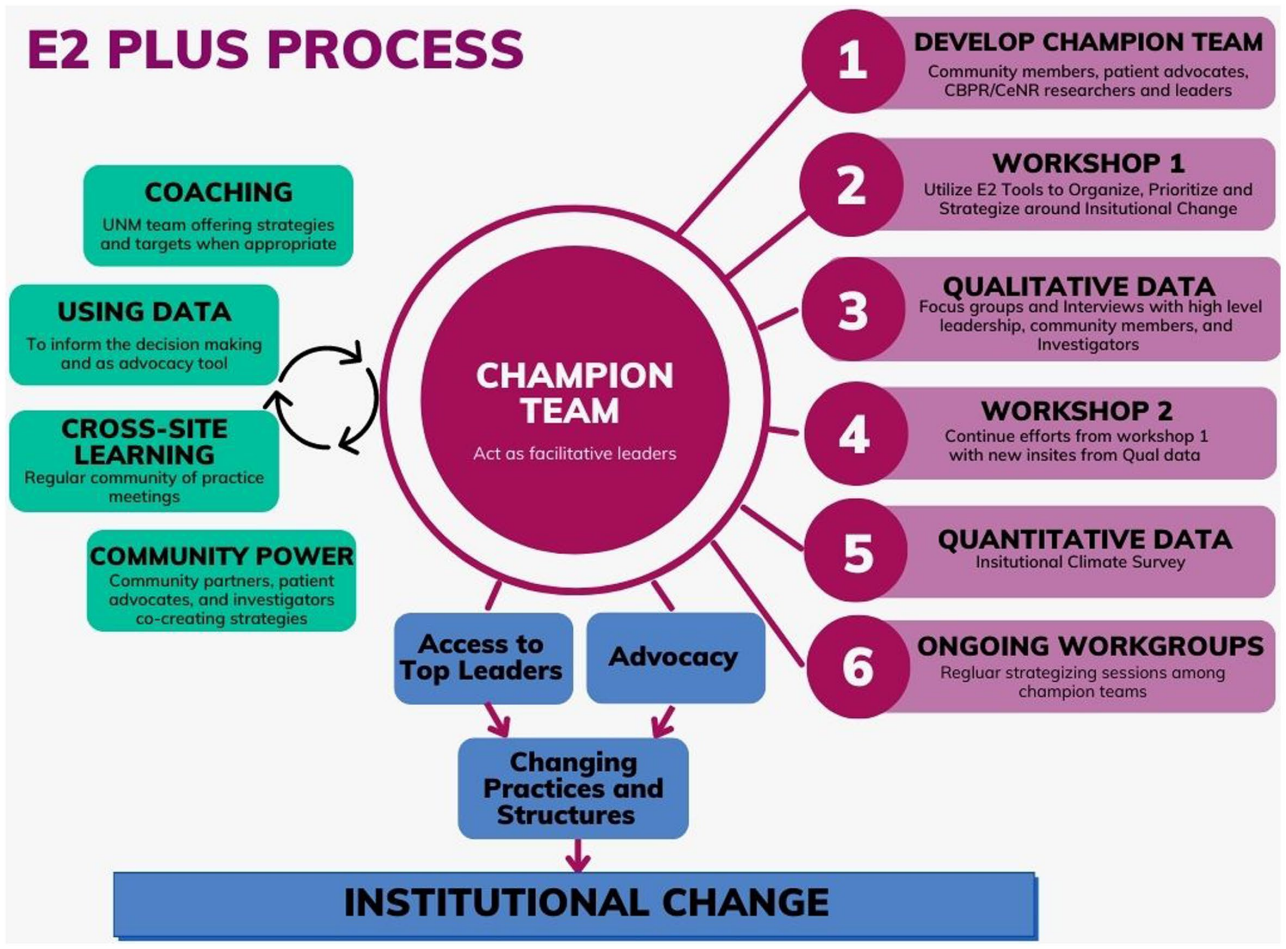
Engage for equity intervention strategies.

As a conduit between funders, institutions, projects, and communities, champion teams are the core target of our engagement intervention as shown in Figure 2, which describes our core intervention strategies and processes. Through coaching, workshops, and ongoing meetings our theory of change proposes that sustained interpersonal relationships between stakeholders and systematic and contextual analyses of power can build the trust necessary to stimulate the collective organizing needed to transform inequitable structural conditions both in and outside of academic institutions.

While institutional rules, policies and norms shape the arena for collaboration, theories and evidence from public administration, public policy, and organizational development demonstrate that adopting *facilitative leadership practices* stimulates transformative action (s) and creative problem solving practices that generate successful policy outcomes and effective implementation of solutions for complex problems (52, 53).

Less pronounced than traditional, top down command and control management, facilitative leadership practices are performed collectively and are shared among groups of representatives appointed by key partners (54–56). Best practices suggest that representatives should include those with formal power to make decisions, those who can successfully block a decision, those affected by a decision, and those with relevant expertise and experience (57). In our intervention, this includes appointed leaders of community engagement centers, faculty conducting community engaged scholarship, community based organizations, patient advocates, and community and patient representatives who have participated in research. In short, this intervention galvanizes advocates of community engaged research to organize for change within academic institutions.

Successful facilitative leaders play different roles to mobilize for change (58). They convene relevant and affected stakeholders to clarify and emphasize interdependence, align goals, and build interpersonal trust in the face of diverging interests. They facilitate work groups that use adaptive strategies to enhance information sharing and mutual learning. They catalyze innovations that solicit new and sometimes disruptive knowledge to encourage groups to think out of the box. They mediate conflicts between communities, funding requirements, and institutional policies. Finally, they steward the ongoing collaboration by protecting it from external pressures (47, 58).

Facilitative leadership practices have been shown to improve multi-sector collaborative processes, accelerate the dissemination and implementation of solutions, and improve innovations and outcomes in a variety of arenas including health policy (59). Within institutions, these practices have been shown to improve the quality of collaboration between multi-sector groups, they produce more precise and nuanced understanding of complex problems, and they create a common ground for a diversity of stakeholders to communicate with each other and deal constructively with differences (56, 60). Facilitative leadership practices also demonstrate the potential to generate governance and sense-making spaces where communities as systems are able to advocate for more collective control over decisions/actions impacting their lives and health with their institutional partners (61). Finally, and at a systems level, facilitative leadership has also been theorized to enable coordinated implementation and adaptation of solutions and these practices accelerate the diffusion of successful innovations (59).

In this E2 PLUS intervention, data also serves an important role, enabling champion teams to not only characterize problems, but to generate evidence-based solutions that pay heed to institutional priorities and constraints faced by top administrators (62). Additionally, mixed methods institutional assessments also included an analysis of more subtle obstacles such as systematic biases that perpetuate social hierarchies in race, class and gender within AHCs as well as more diffuse norms and discourses that legitimate solutions for health equity rather than others. By understanding institutional barriers and leader priorities, we expect that champion teams are able to create solutions that attend to both top level and bottom up needs and concerns.

The E2 PLUS intervention is also grounded in deep engagement of patients/community members to enhance their own facilitative leadership by elevating their governance power and collective empowerment within the change process (7, 18, 57, 63). A unique aspect of our work is to craft new spaces for stakeholders in institutional academic health centers and a broader system of community stakeholders to recognize and develop shared interests to promote institutional capacity and long-term health equity changes.

Similarly, community of practice meetings between each of the institutions’ champion teams allow for shared learnings from past experiences and learnings from their engagement in this project. These meetings are designed for our partners to compare institutional policies and practices that enable equity-based PCOR/CEnR planning and for champion team representatives to cross-share their successes and challenges in advocating for change within their respective institutions. In the final stages of the grant in May 2023, 6–8 members from each site’s champion teams will come together for a conference to cross-analyze their efforts and plan for their engagement steps ahead.

In sum, complementing an iterative approach, the E2 PLUS design provides continuous input of institutional data, and varying stakeholder voices from workshops, coaching, and multi-institutional learning to promote change. As a result, champion teams are expected to make informed decisions that integrate community voice and build power with broader institutional stakeholders to stimulate change. The primary role of the UNM-CPR team is to act as an external consultant team to facilitate these meetings with evidence-based E2 tools and to provide experience-based coaching where teams had gaps in knowledge or experience. As an outside observer bringing awareness to PCOR/CEnR and creating additional pressure on institutional decision-makers, UNM has entered this space and conversation to stimulate forward movement within the champion teams and leadership within each institution. Using tools grounded in emphasizing collective capacity, community cohesion, and community power, E2 PLUS moves beyond promoting one-to-one relational practices focused on stimulating individual cognitive changes among decision makers, towards strategies promoting rapid cycles of collective analysis and collective action for change. E2 PLUS attends to the organizational and relational sources of social power to both coordinate and advocate for change.

### 3.2. Expected outcomes

As a feasibility study, we expected that coaching, workshops for collective reflection, data analyzing power imbalances and institutional capacity, and cross-site learning, would enable champion teams to adopt facilitative leadership strategies. We expected that our intervention strategies would enhance the capacities of community and academic participants to take on more leadership development, build new membership from other diverse stakeholders who can engage with others to adopt advocacy strategies that enable health equity efforts over time, distribute responsibilities across a wide collaborative network, and become stronger “boundary spanners” between community/patient advocates and academic health center leaders. We specifically supported champion teams to adopt advocacy strategies to influence decision-making by top academic leaders to reform institutional policies, norms and practices that deepen engagement to support health equity efforts (58).

An equally important long-term goal of our intervention has been to increase institutional trustworthiness in AHCs over time in order widen the scope of systemic efforts between AHCs, community stakeholders, and health systems to impact structural determinants of health. Trust in public institutions has been shown to improve other multi-sector collaborative efforts to co-develop and implement policies and programs in health, resource management, climate control, and social policy (22). As a multi-dimensional concept, institutional trustworthiness includes attributes along two major dimensions: good faith and competence (64).

Good faith refers to public beliefs that the institution will act in at the interests of relevant stakeholders because it exhibits values that emphasize promise keeping (integrity) and demonstrates that it cares about place and identity based communities through the development and sustainment of ongoing initiatives (benevolence); the public then perceives that the institution demonstrates a track record for public initiatives that follow rules and priorities co-established by institutions and their institutional partners (compatible incentives). Similarly, competent institutions demonstrate they have the ability and power to bridge multiple interests, such as conducting research that prioritizes relevant solutions for patients and communities. Competent institutions are also perceived to be consistent and predictable enough for institutional community partners to forecast potential outcomes when they join together (64).

## 4. Preliminary engagement findings after year 1

We collected qualitative data in the first year of the study to complete our initial institutional assessment. This included AHC leader interviews, stakeholder focus groups, patient/community dialogues at workshops and at advocacy meetings, and observational field note data. Interviews and focus groups probed into institutional contexts and how and to what extent different partners view health-equity oriented P/CEnR within their CTSA and larger AHC and the possibility for change. Using all data sources, we conducted an initial thematic analysis (65) using ATLAS.ti (66) to organize notes and transcripts into a relational database to assist in coding, searching, and retrieving textual data for each site. We followed standard process evaluation using qualitative iterative data collection and analysis feedback loops with deductive as well as inductive logic. We analyzed data throughout data collection period and research team members independently reviewed the data, following an editing approach to identify preliminary themes. This immersion-crystallization analysis stage identified any data inconsistencies. Champion teams also participated in the co-interpretation of their own data at six-month intervals as a participatory process for greater validity and for enhanced ownership of processes and findings.

Qualitative findings from leader interviews, community/patient and investigator focus groups early in the first year set the stage for analyzing the different contexts of each institution, and at the same time, uncovered shared tensions, showcasing that all could improve their accountability to communities. These tensions ranged from institutions acknowledging the outsize influence of external influences, such as the dominance of basic science and clinical NIH funding, including genomics and precision medicine, to the internal challenges of administrative and financial barriers in post-award, IRB, and other research processes. While leaders and investigators believed they were seeing changes through their equity or anti-bias training efforts, community members and patient advocates often talked about their continued experience of exclusion, with insufficient resources for community engagement. Even with successes in diversity, equity, and inclusion (DEI), respondents felt these were not connected enough to community and patient engagement. Many believed that “policies are there not to protect the community, but to protect the university.”

These tensions manifested differently at each institution. Participants from Morehouse, for example, more than the other two institutions, valued that equity and community engagement were in the DNA and history of the institution, yet still identified the challenge of insufficient resources to realize equity goals, including that too few people had the CBPR expertise needed in order to grow engagement throughout the institution. Stanford participants, on the other hand, recognized the paradox of being from a prestigious national research institution in basic science and medicine, yet expressed concern for the lack of access in both clinical care and research involvement for community members. Fred Hutchinson/University of Washington Cancer Consortium, in particular, felt the tensions of movement forward on DEI yet with insufficient connection to community engagement.

As the E2 PLUS intervention progressed with the UNM team providing workshops and coaching, using the River of Life, Visioning with the CBPR Model, and a synthesis of the qualitative data at each site for their own understanding and interpretation, champion teams identified targets and advantage points for change. In the two workshops, participants identified institutional barriers including a lack of financial transparency with communities; lack of timely payments to patient advocates and community organizations; institutional review board’s lack of understanding the nuances of PCOR/CEnR; insufficient collaboration among PCOR/CEnR internal efforts; and insufficient PCOR/CEnR training for investigators and community members/patient advocates. Even with variation in institutional readiness for PCOR/CEnR, by the middle of the first year, champion teams had identified working groups and expanded advocacy through their access to top leaders for change, such as, (1) pursuing a new Office of Patient Engagement at Fred Hutchinson; (2) challenging inflexible institutional review board processes at Stanford; and (3) enhancing strategies for expanded community diversity within Morehouse’s premier Prevention Research Center Community Coalition Board.

In sum, even after 1 year of E2 PLUS, preliminary findings show enhanced effectiveness of champion teams to reach our goal, becoming stronger facilitative leaders and boundary spanners between top leaders and institutionally-connected community and patient partners. Champion teams also used quantitative and qualitative assessments to enhance their ongoing advocacy for changes for strengthening community and patient engagement. Patient and community voices have been given more attention at the leadership level, though the work continues. As one community member has said, “they have to make sure the community feels that they really want to hear their voices and not are just putting on a show. They need to give them appropriate compensation for the engagement of their time and their expertise which is in fact of great value.”

## 5. Discussion

Adapting Popay’s (21) limiting framework, the initial results indicate that the combination of mixed methods data analysis, workshops, and ongoing champion team coaching uncovered multiple forms of power imbalances that constrain successful systemic engagement in AHCs. Multi-method data analysis offered community and patient stakeholders a clearer picture of how authority was organized in each academic health center. Important aspects of authority were revealed in our preliminary analysis including defining what departments make decisions on how to allocate financial resources, how bureaucrats implement contracting and post-award grant processes, and which stakeholders determine IRB processes. Workshops and ongoing meetings crystalized how fragmentation of multiple engagement centers and initiatives was its own barrier in transforming institutions, with ongoing reflections uncovering how external funding and institutional leaders shape the substantive direction of research that influences the adoption of some health equity solutions as opposed to others.

Other, less visible, forms of power constrain collaborations in important, yet more subtle, ways. For example, structural power, which is invisible and embedded in broader social institutions, limits the capabilities of AHCs and their multi-sector partners to generate transformational health equity changes that address root causes of disease. Structural power generates and sustains social hierarchies of class, gender, and race/ethnicity through the distribution of resources, opportunities, and social status of groups (38). Examples of these structural constraints include when academic institutions make few investments in strengthening organizations of disadvantaged people to build their collective capabilities for long-term change and when they continue to replicate structural inequities through policies and practices that are institutionally racist and gendered. Patterns of structural racism were often cited as a key barrier at each site.

Productive power operates through diffuse social discourses and practices that legitimate some forms of knowledge, while marginalizing others (38, 67). Related to CEnR research, there is evidence from these sites that AHCs present barriers to meaningful community engagement research and action due to epistemic biases in what constitutes acceptable research, neoliberalist tendencies to generate research dollars that support the status quo, gendered norms, and colonial racist defaults (67). For example, many tenure and promotion requirements and institutional commitments are not currently organized to support effective community and patient engagement (68).

The initial results also indicate that champion teams had to be what Bryson and colleagues call “structurally ambidextrous” to manage multiple tensions in reforming policies and procedures (59). These tensions included the need to juggle institutional stability versus change, using lateral relationships to challenge hierarchical processes while still respecting the authority of university leaders, using formal versus informal networks to advocate for change, and using existing forums versus creating new ones for CeNR health equity (59).

Congruent with other findings in public administrative research (59, 69), our initial results suggested that champion teams made calculated decisions to invest their initial efforts in reforming organizational practices that have a direct impact on community engagement while acknowledging that other policies and norms are likely to continue reinforcing structural and epistemic power imbalances. Our preliminary results also demonstrate that the intervention helped champion teams generate change strategies based on lateral relations between community/patient stakeholders and formal partners, through new forums enabled through workshops, and by collectively reflecting on data that generated more power sharing to stimulate change strategies. Despite acknowledging the broader systemic issues, initial findings suggest that as teams moved to implement changes in existing processes like contracting and grants and IRB administration, champion teams also had to adapt to the hierarchies, by soliciting change from leaders and formal networks, with less power sharing, so that changes could be enacted. Future research needs to explore what kinds of ambidexterity are necessary to address these and other power imbalances.

## 6. Conclusion

The UNM team recognizes the dedication and forward movement of individuals, departments, CBOs, patient advocates and other stakeholders to improve equity-based PCOR/CEnR/CBPR that occurred in the past and independently occurred during this intervention. The purpose of the E2 PLUS intervention was to enhance the existing efforts and to inspire new ones with organizing and power-sharing with community members and patients.

Overall, the initial analysis demonstrates that champion teams formed quickly, they used data and workshops to plan for targets of change, and they were successful in mobilizing for policy and practice changes. Overall, the intervention shows promise in supporting champion teams through workshops, coaching, and data analysis to become agents of change in another and perhaps, deeper way. The initial results suggest that E2 PLUS provides a venue for diverse stakeholders to create greater connectivity between systems of academic community engagement and committed stakeholders: (1) to establish opportunities for collective decision making and forming wider alliances; (2) to identify and act on existing power dynamics that undermine the capabilities of diverse groups in developing collaborative solutions that promote health equity; and (3) to create new “sense making spaces” (61) in which participants collectively reflect on the stigmatizing discourses and inequalities that sabotage true health equity reform, while developing, newer, longer-term narrative strategies in the hope of prompting deeper changes.

## Data availability statement

The raw data supporting the conclusions of this article will be made available by the authors, without undue reservation.

## Ethics statement

The studies involving human participants were reviewed and approved by University of New Mexico Health Sciences Center (HRRC: # 21-320). Written informed consent for participation was not required for this study in accordance with the national legislation and the institutional requirements.

## Author contributions

SS-Y and NW conceived the idea and theory of change and drafted the manuscript. SS-Y, PA, AG, KM, NW, and CA collected and analyzed the data. PA created the logic model. AG created the theory of change figure. PA and ED critically reviewed the manuscript. All authors contributed to the article and approved the submitted version.

## Funding

This research was funded by the Patient Centered Outcomes Research Institute Engagement (PCORI) Award, “Engage for Equity: Advancing Research Support for Institutional, Patient and Stakeholder Partnering”. PCORI Eugene Washington Engagement Award ##21068.

## Conflict of interest

The authors declare that the research was conducted in the absence of any commercial or financial relationships that could be construed as a potential conflict of interest.

## Publisher’s note

All claims expressed in this article are solely those of the authors and do not necessarily represent those of their affiliated organizations, or those of the publisher, the editors and the reviewers. Any product that may be evaluated in this article, or claim that may be made by its manufacturer, is not guaranteed or endorsed by the publisher.
